# Right-sided "trapdoor" incision provides necessary exposure of complex cervicothoracic vascular injury: a case report

**DOI:** 10.1186/1757-7241-17-46

**Published:** 2009-09-24

**Authors:** Boris Kessel, Itamar Ashkenazi, Isaak Portnoy, Dan Hebron, Dani Eilam, Ricardo Alfici

**Affiliations:** 1 Trauma Unit, Hillel Yaffe Medical Center, Hadera, Israel; 2 Surgery B Department, Hillel Yaffe Medical Center, Hadera, Israel; 3 Vascular Surgery Department, Hillel Yaffe Medical Center, Hadera, Israel; 4 Interventional Radiology Unit, Hillel Yaffe Medical Center, Hadera, Israel

## Abstract

Combined cervicothoracical vascular traumas are very uncommon, mostly resulting from penetrating injuries. These injuries are accompanied with very high morbidity and mortality rates. In this manuscript we present a case of hemodinamycally unstable trauma patient whose major injury was penetrating trauma of both cervical and mediastinal major vessels. The standard surgical approach of median sternotomy and neck incision was insufficient, and the patient's instability forced the authors to improvise previously not described right-sided trap-door thoracomy. Incorporation of such incision in the surgical arsenal may be very effective in selective cases

## Introduction

Combined cervicothoracical vascular traumas are uncommon, mostly resulting from high energy penetrating injuries [1,2]. These injuries are diagnostically and therapeutically challenging even for a very experienced multidisciplinary trauma team. Patients suffering from hemodynamic instability are taken immediately to the operating room. In presence of the right sided vascular injuries, the common surgical approach is via a median sternotomy [3]. Combination of anterolateral thoracotomy, partial sternotomy and left infra or supraclavicular incision described as "trap-door" thoracotomy is rarely performed since it is time-consuming and results in multiple fractures [4],. We present here a case of hemodinamycally unstable trauma patient whose major injury was penetrating trauma of both cervical and major mediastinal vessels. An improvised right sided "trap-door" thoracotomy was necessary to achieve vascular control and reconstruction.

## Case description

A twenty eight years white male was admitted to the trauma resuscitation area following a gunshot assault. On admission the patient was agitated. Vital signs revealed: blood pressure of 120/65, heart rate of 110 per minute and oxygen saturation of 94% on oxygen mask; and respiratory rate of 20 per minute. Physical exam revealed an entry wound located at the posterior aspect of the right shoulder. The exit wound was located at the left side of the neck, posterior to left sternocleidomastoid muscle. A large right upper chest wall hematoma, extended to the neck was found which was not pulsating. Pulse on both carotid arteries was intact. There was no active bleeding from both entry and exit wounds. The right upper extremity was pale and swollen, with no palpable pulse. Breath sounds were equal and of good intensity bilaterally. The rest on physical examination was unremarkable. The patient was treated with immediately intubation and mechanical ventilation. Intravenous bolus of crystalloids was started. Portable chest x-ray in the trauma room revealed no pneumothorax.

During the initial stay in the trauma resuscitation area, the patient became hemodynamically unstable. Despite fluid administration, he developed tachycardia, up to 136 per minute, and blood pressure dropped to 80/44. The cervical hematoma seemed to be increasing in size. Following this deterioration, the patient was immediately taken to the operating room.

**Figure 1:**
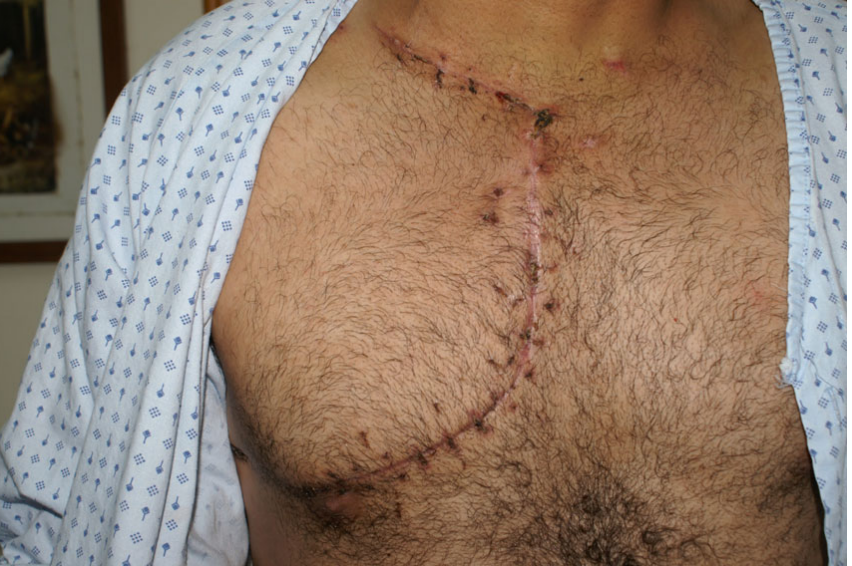
**Displays how the incision was extended by a right anterolateral thoracotomy, performed through the third intercostal space**.

On surgery, due to clinical impression of injury to the distal subclavian artery, a right supraclavicular incision was performed first. Following incision of the platysma and division of the right sternocleidomastoid muscle, significant hemorrhage appeared in the surgical field that was temporally controlled by direct digit pressure application. Recognizing this to be hemorrhage possibly arising from major vessels in Zone I of the neck, a full mid sternotomy was performed to allow proper exposure and vascular control. However, even following sternotomy, the athletic habitus of the patient did not allow delineation and approach to the major sources of bleeding. The incision was extended by a right anterolateral thoracotomy (Fig 1), performed through the third intercostal space. This right-sided "trapdoor incision" allowed adequate exposure and proximal control of the mediastinal vessels. Tears of both the right common carotid artery and the right innominate artery were found at their confluence. The right jugular vein was injured as well. Repair of the arterial injury was achieved by placing a graft patch modified from a collagen coated knitted polyester vascular prosthesis (Silver Graft, Datascope, Montvale, USA). The vein was repaired by primary suture of the tear. At this stage of operation the patient was hypothermic (34°Celsius) and there was clinical evidence of coagulopathy. We decided not to continue with the exploration of the distal subclavian artery. The operation was promptly terminated by packing the neck and upper mediastinum, followed by temporary closure of the wounds. Overall, the operation lasted 2 hours and 47 minutes.

**Figure 2:**
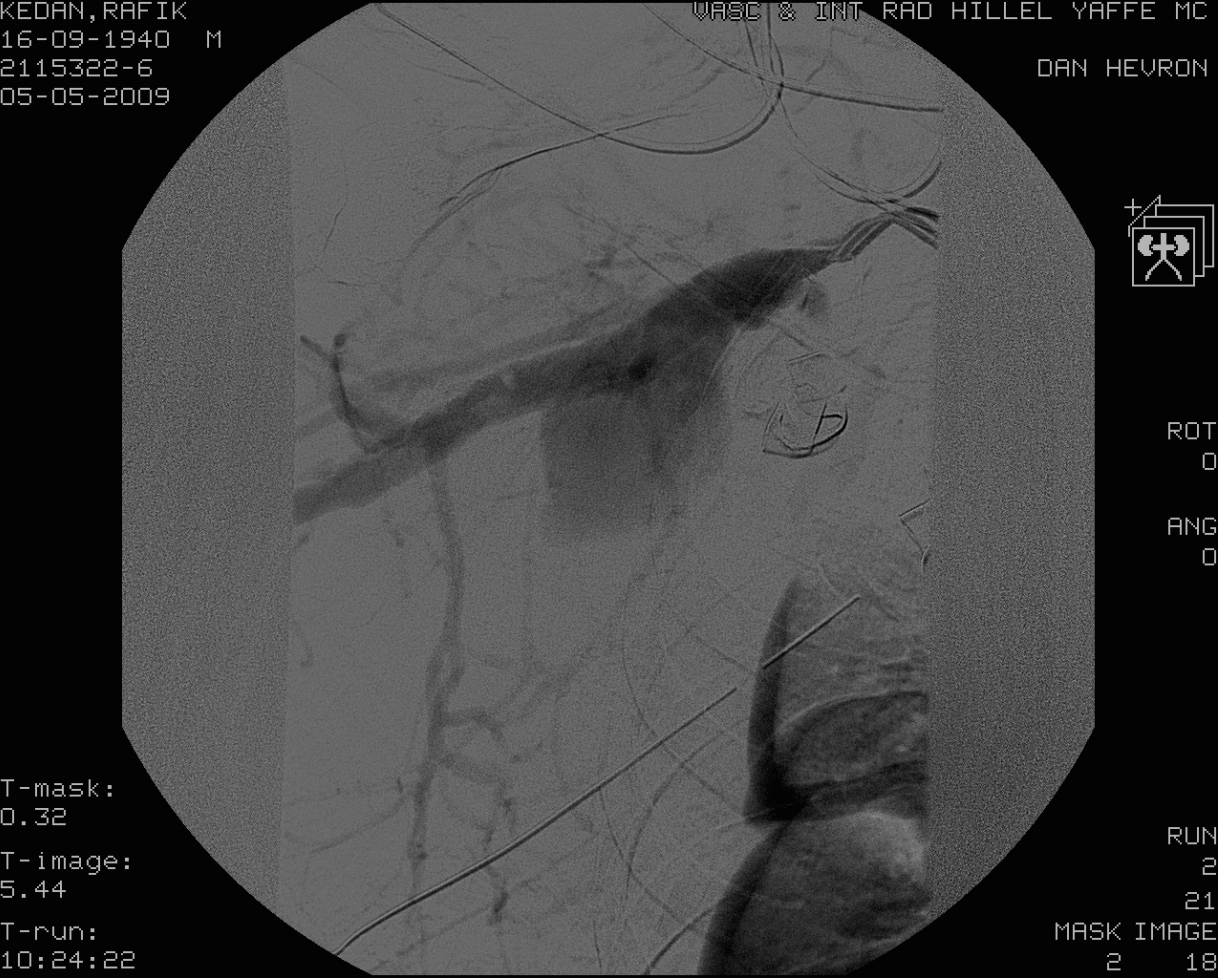
**This figure illustrates massive extravasation from to tear located at the distal part of the right subclavian artery**.

**Figure 3:**
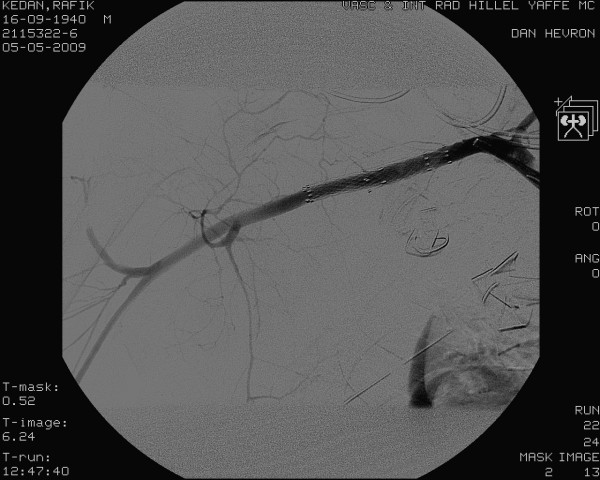
**No signs of contrast extravasation were demonstrated after the procedure**.

The patient was transferred to the postoperative recovery unit where he was rewarmed and resuscitated with blood and fresh frozen plasma. Following 4 hours, he was hemodynamically stable but still dependent on fluids. Coagulation tests returned towards normal values. The right upper extremity was ischemic. At this point, he was transferred to the angiography suite. We assumed that the patient had an injury of subclavian artery which was not dealt with during the initial operation. Via a right groin approach, neck vessels and selective right subclavian artery angiography was performed." This revealed massive extravasation from to tear located at the distal part of the right subclavian artery (Fig 2). Three Fluency covered stents (Bard Corporate, Murray Hill, NJ) were placed and no signs of contrast extravasation were demonstrated after the procedure(Fig 3). Blood flow to right upper extremity was restored.

The patient was transferred to the Intensive Care Unit. Twenty four hours later he was reoperated. He underwent depacking of the neck and upper mediastinum and the trap-door incision was closed in the usual fashion. At postoperative day three he was extubated and two days later he was transferred to the surgical ward. Due to accumulation of pleural blood which would not drain following reinsertion of a chest tube, a video-assisted thoracoscopy was performed at postoperative day ten and significant amounts of blood clots were evacuated." The rest of hospital stay was uneventful and the patient was discharged home after three weeks.

## Discussion

Penetrating injuries of the thoracic great vessels are associated with high morbidity and mortality. Many patients die on the scene from massive hemorrhage. Mortality is significant even in patients who survive the initial period of injury and are alive on their admission to the hospital. Demetriades et al. report overall mortality of 34.2% in patients suffering from subclavian and axillary artery injuries [5]. Patients with innominate artery injury usually do not survive to arrive at the hospital. There are only few case reports that describe patients who were treated for combined common carotid and innominate artery injuries [6,7]. In these, the injury was located to the left hemithorax, unlike our patient in whom these vessels were injured on the right side.

Management of major vascular injuries in the base of the neck is complex. If the patient is stable, the first diagnostic step should be cervical and chest CT-angiography. CT angiography provides the necessary information regarding the spectrum of vascular, mediastinal and other injuries. This information is crucial, allowing proper decision making concerning the therapeutic plan. In the hemodynamically stable patient, significant vascular injury may be treated with endovascular stenting.

If the patient is hemodynamically unstable, he/she should be taken immediately for surgery. The selection of the incision depends on mediastinal structures that need to be explored during the surgery. In the case of a clinical suspicion for right common carotid artery injury, the oblique incision along the anterior border of the sternocleidomastoid muscle should be performed [8], with extension to median sternotomy, if proximal control of the injury is difficult. This surgical exposure usually provides excellent approach to other injuries of the innominate and common carotid artery. In our patient, this proved to be insufficient. The patient instability forced us to improvise a right "trap-door" thoracomy. Defined by some as being "obsolete"[5] this incision facilitated to achieve fast control of bleeding in this patient.

## Conclusion

In selective cases median sternotomy does not provide adequate exposure of the mediastinal great vessels. Incorporation of right sided trapdoor thoracotomy may be very efficient in complex cervicothoracic trauma.

## Consent

Written informed consent was obtained from the patient for publication of this case report and accompanying images.

## Competing interests

The authors declare that they have no competing interests.

## Authors' contributions

BK was the case manager and was the main writer to draft the manuscript. IA and DH helped draft the manuscript and added significant revisions. IP, DE and RA read the manuscript and added significant revisions. All authors discussed the details of the case, implications of the case and commented on the manuscript at all stages. All authors read and approved the final manuscript.
